# Evaluation of the impact of oximeter averaging times on automated FiO_2_ control in routine NICU care: a randomized cross-over study

**DOI:** 10.3389/fped.2023.1240363

**Published:** 2023-09-22

**Authors:** J. Janota, J. Dornakova, V. Karadyova, R. Brabec, V. Rafl-Huttova, T. Bachman, M. Rozanek, J. Rafl

**Affiliations:** ^1^ Department of Neonatology, Motol University Hospital, Prague, Czechia; ^2^ Department of Biomedical Technology, Faculty of Biomedical Engineering, Czech Technical University in Prague, Kladno, Czechia

**Keywords:** oxygen control, automated oxygen control, pulse oximetry, neonatal, SpO_2_ targeting

## Abstract

**Objective:**

Changes in oximeter averaging times have been noted to affect alarm settings. Automated algorithms (A-FiO_2_) assess FiO_2_ faster than oximeter averaging, potentially impacting their effectiveness.

**Methods:**

In a single NICU routinely using 15 fabian-PRICO A-FiO_2_ systems, neonates were randomly exposed to SpO_2_ averaging time settings switched every 12 h among short (2–4 s), medium (10 s), and long (16 s) oximeter averaging times for the entire duration of their A-FiO_2_ exposure. Primary endpoints were the percent time in the set SpO_2_ target range (dependent on PMA), SpO_2 _< 80%, and SpO_2 _> 98%, excluding FiO_2_ = 0.21.

**Results:**

Ten VLBW neonates were enrolled over 11 months. At entry, they were 17 days old (IQR: 14–19), with an adjusted gestational age of 29 weeks (IQR: 27–30). The study included data from 272 days of A-FiO_2_ control (34% short, 32% medium, and 34% long). Respiratory support was predominantly non-invasive (53% NCPAP, 40% HFNC, and 6% NIPPV). The aggregate SpO_2_ exposure levels were 67% (IQR: 55–82) in the target range, 5.4% (IQR: 2.0–10) with SpO_2 _< 80%, and 1.2% (IQR: 0.4–3.1) with SpO_2 _> 98%. There were no differences in the target range time between the SpO_2_ averaging time settings. There were differences at the SpO_2_ extremes (*p* ≤ 0.001). The medium and long averaging were both lower than the short, with the difference larger than predicted. Multivariate analysis revealed that these findings were independent of subject, ventilation mode, target range, and overall stability.

**Conclusions:**

This A-FiO_2_ algorithm is effective regardless of the SpO_2_ averaging time setting. There is an advantage to the longer settings, which suggest an interaction with the controller.

## Background

Continuous monitoring of oxygenation (SpO_2_) by pulse oximetry is the standard of care for preterm infants receiving supplemental oxygen. Relatively modest changes in oxygen saturation exposure are associated with a marked impact on outcomes (1–3). Neonatal oxygenation is unstable, and nurses struggle to manage SpO_2_ within prescribed target ranges. Compliance is routinely only 50%, and excessive hyperoxemia and hypoxemia are prevalent in routine care (4–6). An essential part of oxygenation management is temporarily increasing inspired oxygen (FiO_2_) to address intermittent hypoxemic episodes. Importantly, once the instability has resolved, a prompt return to baseline FiO_2_ is needed to reduce potential hyperoxemia.

Following decades of development, newer neonatal ventilators offer closed-loop titration of FiO_2_ based on the continuous monitoring of SpO_2_ (A-FiO_2_). A-FiO_2_ systems have been consistently shown to be effective (7). Nevertheless, while there are dozens of positive studies, the evaluative populations are narrow. Most of the studies include a few systems, and the control algorithms are quite different (8). Further, nearly all of the studies have a short physiological cross-over design, which is not necessarily reflective of the routine practice of weeks of supplemental oxygen. There are a few studies of the extended use of a few systems (9–12) and the subtleties of A-FiO_2_ settings in routine care (13, 14).

Most commercial oximeters used in neonatal care offer clinicians the option of adjusting the averaging time to mitigate physiologic and measurement noise. Even the shortest averaging times, however, reflect multiple peripheral pulses. While other internal oximeter software attempts to reduce the impact of artifacts, it remains a common problem. In contrast, fidelity is clearly lost with averaging, and analyses confirm that averaging time affects the monitored depth and duration of reported exposure (15, 16). In all A-FiO_2_ systems, the SpO_2_ averaging time is much slower than the frequency of SpO_2_ assessment and much slower than the rate of FiO_2_ adjustment in some systems. Nevertheless, there are no thorough evaluations of the interaction of the set SpO_2_ averaging time setting with the performance of A-FiO_2_.

Motol University Hospital has a large tertiary neonatal care center, and 15 A-FiO_2_ systems have been used routinely since January 2019. Although it is known that shorter averaging times are more accurate, the subjective impressions of the staff regarding an optimal approach to setting the SpO_2_ averaging time have been inconsistent. The aim of this study was to explore the impact of averaging time on SpO_2_ control during A-FiO_2_.

## Methods

This is a single-site randomized cross-over study in which the SpO_2_ averaging time setting switched every 12 h over the course of routine care. The study was approved by the institution's Bioresearch Ethics Committee (Reference number EK-1548/21, 1 December 2021, Ethics Committee of the University Hospital Motol and Second Faculty of Medicine, Charles University, Prague). Written parental informed consent was required and received before enrollment. The study was prospectively registered (ClinicalTrials.gov, NCT05274386).

Only fabian-PRICO A-FiO_2_ systems (Vyaire Medical, Mettawa, USA) were used in this study. In these systems, A-FiO_2_ control is available for all ventilation modes (HFOV, CMV, NIPPV, NCPAP, and HFNC). The PRICO A-FiO_2_ (**PR**edictive **I**ntelligent **C**ontrol of **O**xygenation) system monitors SpO_2_ every second using an integrated Masimo pulse oximeter. Based on the weighted average of these data, an adjustment in FiO_2_ is made every 30 s if warranted. Within the set target range, this adjustment is ±1% toward the midpoint. Outside the target range, the adjustment varies by ±1%–10%. The amount of adjustment is based on a proprietary algorithm that takes into account the depth and trajectory of the predicted response to changes in oxygen. In addition, when SpO_2_ moves outside the target range, an initial adjustment is made and the 30-s period is reinitiated. Under certain conditions (SpO_2_ dropout, exceeding operator-set parameters), the system falls back to manual control at a FiO_2_ level previously specified by the clinician. The system returns to A-FiO_2_ control when the condition resolves or with operator reactivation.

Written Case Report Forms (CRF) for each subject captured the demographic and baseline information, as well as the exact time and average-time setting, relevant study events, and reason for exit from the study. Ventilator system data were collected concurrently from a bedside PC using purpose-coded Matlab software (MathWorks, Natick, USA). These ventilator data were captured every 2 s and included the measured SpO_2_, set FiO_2_, set SpO_2_ control range, and set ventilation mode. CRFs and digital data were concurrently reviewed by the investigators, and potentially spurious information was evaluated. These data were merged with the averaging settings and gestational age from the CRFs into an analytical database.

The SpO_2_ averaging time setting was changed every 12 h (10 am, 10 pm). Subjects were alternated between three averaging times (2–4, 10, and 16 s, or short, medium, and long, respectively). The sequence was assigned a predetermined random order, different for each subject, and integrated into the subject-specific CRF. The assigned sequence was composed of balanced blocks, so every subject was exposed to all three average settings twice every 3 days. All other aspects of care were standard according to the unit policy and clinical judgment. The unit policy included tiered target ranges/alarms based on postmenstrual age (<29 weeks: 88%–92%, 29–33 weeks: 90%–94%, 34–36 weeks: 92%–96%, >36 weeks: 95%–98%, with alarms set at 1% outside the target range).

With informed consent, infants were eligible for enrollment if their birth weight was <1,500 g, they had no congenital anomalies, and they required respiratory support and supplemental oxygen at 2 weeks of age. The latter is consistent with unit policy, as A-FiO_2_ is not routinely used in the acute phase following birth. Subjects were excluded from the study if they were weaned from supplemental oxygen, transferred, or completed 30 days of intervention.

All endpoints were prospectively defined for the study, except as indicated. The primary endpoints were compliance (percentage of time in the intended SpO_2_ target range) and safety (SpO_2_ < 80% and SpO_2_ > 98%). Secondary endpoints included time above and below the target range. Periods with SpO_2_ higher than the target range and FiO_2 _= 0.21 were included in the target range compliance and excluded from time above the target range. Other descriptive parameters were also collected. Sensitivity analyses were prescribed to evaluate the impact of covariables. These covariables were set: target range, ventilation mode, and, added on a *post hoc* basis, stability. Stability was assessed based on the mean time in the SpO_2_ target range in each 3-day block. It was categorized as stable if greater than the median time (67%) for all subjects in the study or less stable if lower.

The power analysis was based on the safety endpoints and specified differences that were small but considered potentially clinically relevant. Based on other trials, we nominally expected a mean SpO_2 _< 80% of 2% ± 3% and a mean SpO_2 _> 98% of 4% ± 6%. We determined that a difference for SpO_2 _< 80% of 1.5% and a difference for SpO_2 _> 98% of 3.0% would be detected with more than 80% power, an alpha of 0.05, and a total of 50 measurements in each averaging group. These differences were larger than expected based on the change in fidelity from averaging SpO_2_ (13). The projected rate of enrollment, considering the likely unit census, two data collection systems, and staff resources, was one subject per month. A minimum of 10 neonates was considered an acceptable sample size. With 10 subjects, 50 measurements would be achieved with an average of 2 weeks of intervention. Thus, the study was designed to continue until at least 10 subjects were enrolled with at least 50 SpO_2_ paired averaging time measurements.

To address potential carry-over between averaging epochs, the first and last 10 min of each epoch were excluded from all analyses. A general linear model (ANOVA) was used for each of the three independent primary endpoints, with independent (explanatory) covariables. These included SpO_2_ set-average as a fixed variable and four random control covariables (target range, mode of ventilation, stability, and subject). If needed, the dependent variables were to be log-transformed to address a lack of normality (Shapiro–Wilk), which was the case. The effect size was determined with the shortest averaging time as the baseline. For consistency, descriptive data were presented as median and IQR, regardless of normality. *p* < 0.05 was considered statistically significant. Covariables needed to be cross-tabulated to explore potential clinical significance if they were relevant to the significance of the SpO_2_ averaging time setting. All statistical comparisons utilized XLSTAT 11.5 (Lumivero, NY, USA).

## Results

The study began in February 2022 and ended in December 2022, when a minimum of 10 subjects had completed the study. During this period, 11 infants met the enrollment criteria. In total, the parents of 10 infants were approached for informed consent when data collection systems were available. Most subjects were ELBW infants aged less than 3 weeks at enrollment. Most had been intubated before entry, but all were being supported on NCPAP at enrollment. Further details of the subjects are listed in Table 1.

**Table 1:** Baseline study population details.

*n*	10
Gender (% male subjects)	60%
Birth weight (g)	870 (728–989)
Gestational age at birth (weeks^days^)	26^6^ (25^6^–28^3^)
Age at entry (days)	17 (14–19)
Weight at enrollment (g)	985 (825–1,084)
PMA at entry (weeks^days^)	29^2^ (27^6^–30^6^)
Previous ventilation mode; CMV/HFO/NCPAP/HFNC	6/0/10/0
Days of CMV prior to enrollment	1.0 (0.0–4.4)
Max FiO_2_ prior to entry (%)	35 (35–39)
Vent mode at entry (NCPAP %)	100%

PMA, birth gestational age plus age at entry; CMV, conventional mechanical ventilation, presented as median (IQR) or percentage.

Details of the subjects’ progress during the study period are provided in Table 2. Most were studied for 30 days, and the data reflect a total of 272 days (545 measurements) of A-FiO_2_ control. Respiratory support was predominantly provided by either NCPAP or HFNC at low FiO_2_ levels, with the target range set at 90%–94% SpO_2_. The duration of exposure to the three set SpO_2_ averaging times was similar, with only 6 out of 545 periods set incorrectly. The balance of the difference was due to terminating enrollment in the middle of the 3-day balanced block. The periods during the study when A-FiO_2_ was disabled were quite short [3 min/12 h (IQR: 2–6)]. However, it was approximately a minute longer during the short averaging periods than during the medium and long averaging periods (*p* < 0.001). No adverse effects/events related to A-FiO_2_ were reported.

**Table 2:** During study intervention.

*n*	10
Vent mode during study (NCPAP/HFNC/other)	53%/40%/7%
TR entry (88–92/90–94/92–96)	5/5/0
TR in the study (88–92/90–94/92–96/95–98)	12%/74%/14%/0.3%
FiO_2_ at entry (%)	27 (23–29)
FiO_2_ at exit (%)	23 (21–24)
Days set-average (s/m/L)	183/177/185
Days in study	30 (28–30)
A-FiO_2_ off (min/day)	3.4 (1.7–6.3)
Exit (30d/no O_2_/transfer)	6/3/1

Other include CMV, HFO, set-average s/m/L = short, medium, long, presented as median (IQR), percentage, or count.

The primary endpoints of SpO_2_ exposure are listed in Table 3. The subjects spent two-thirds of the time in the target range, which was nearly identical to the three averaging times. However, the set SpO_2_ averaging time significantly affected the time at the two SpO_2_ extremes. SpO_2_ was higher during the short averaging time than during the longer averaging time. During the short averaging periods, the mean time with SpO_2_ > 98% was 1.8%, but it was about half that during the other two average-time settings (41% lower, *p* = 0.001 medium averaging, and 51% lower, *p* < 0.001 during long averaging). During the short average periods, the mean time with SpO_2 _< 80% was 6.5%. It was 18% lower during medium averaging (*p* = 0.08) and 31% lower (*p* = 0.003) during long averaging. The percent times below the target range were nearly identical [short: 17% (IQR: 11–26), medium: 17% (9–25), and long: 17% (10–26)]. There was a small difference in the percent times above the target range [short: 13% (IQR: 7–20), medium: 11% (6–19), and long: 12% (7–20), *p* = 0.025].

**Table 3:** Effects of set SpO_2_ averaging time settings: primary endpoints.

Percent time	Short 2–4 s	Medium 10 s	Long 16 s	*p*
SpO_2_ target range	67 (55–79)	67 (54–83)	68 (55–83)	ns
SpO_2_ > 98%	1.8 (0.7–3.6)	1.1 (0.3–2.7)	0.9 (0.2–2.9)	<0.001
SpO_2_ < 80%	6.5 (2.8–11.1)	5.3 (1.8–9.5)	4.5 (1.5–9.4)	<0.001

The target range varied depending on gestational age. Time above the target range with FiO_2_ = 0.21 was included in the target range and excluded at SpO_2_ > 98%. The *p*-values represent the difference between the three averaging times.

The analysis controlled for differences between subjects, ventilation modes, target ranges, and stabilities. Nevertheless, the set SpO_2_ averaging time in the multivariate analysis was independently significantly related to differences in SpO_2_ extremes. Not surprisingly, all of these control parameters were also significantly related to target range compliance and exposure to severe hypoxemia and hyperoxemia. The effectiveness of A-FiO_2_ in the 3-day stable and less stable cohorts offers some insight into its performance. For the two stable cohorts, time in the assigned target range was 82% (73–90) vs. 55% (48–62), time with SpO_2_ < 80% was 2.2% (1.0–4.2) vs. 9.7% (6.6–14), and time with SpO_2_ > 98% was 0.9% (0.3–2.2) vs. 1.8% (0.5–3.8), *p* < 0.001 for all three settings.

## Discussion

Using a cross-over design, we evaluated the impact of the set SpO_2_ averaging time during 39 weeks of routine use in 10 preterm infants. While the differences were small, we found that the shortest of the three averaging times resulted in an increase in time at SpO_2_ extremes but no difference in the times within the intended SpO_2_ target range. This difference was independent of subject, target range, mode of ventilatory support, and stability.

The reported differences suggest that the set SpO_2_ averaging time affects the FiO_2_ control algorithm. Based on the arithmetic of averaging, one would expect that a shorter averaging time would detect more episodes of fluctuations away from the target range with more extreme nadirs but of shorter duration. How this effect might impact the time above or below specific SpO_2_ thresholds is less obvious. The impact of these averaging effects on SpO_2_ monitoring in infants has been studied. In two reports, Vagedes et al. evaluated healthy infants with apnea and children being assessed for sleep apnea (16, 17). They documented the predictability of the effect of a set SpO_2_ averaging time on desaturation. They also reported that longer averaging times result in an inaccurate decrease in the burden of hypoxemia (area under the curve). McClure and colleagues reprocessed 24 days of 2–4-s SpO_2_ data to reflect SpO_2_ averaging of 8 and 16 s (15). They aimed to determine the trade-off between averaging, alarm delay, and alarm settings in the NICU. They reported that the percent time decreased by 4% and 7% (8 and 16 s average, respectively) for hypoxemic events (SpO_2_ < 70%). For hyperoxemic events (SpO_2 _> 98%), they reported that the percent time increased by 1% for 8 s and decreased by 8% for 16 s. In contrast, we found that the impact of averaging time settings was 5–10 times greater. This suggests that the differences we reported are primarily the result of an interaction with the A-FiO_2_ control algorithm. Importantly, the effect we reported was independent of infant stability, ventilation mode, and target range, suggesting consistency across clinical conditions. We can only speculate as to the cause. We ruled out the impact of SpO_2_ dropouts in our study because the time for A-FiO_2_ fallback was very short, although it may be relevant in other situations. This suggests that the set averaging time affects the processing of the second-to-second data by the proprietary predictive equation. However, we cannot exclude a lack of comparability of the McClure data, as the decrease in the area under the curve predicted by Vagedes, albeit in a less comparable population of healthy infants without respiratory or oxygen supplementation, is similar to our findings for hypoxemia.

Short-term cross-over studies may have a selection bias. Our study did not evaluate A-FiO_2_ performance compared to manual control. However, this device has been evaluated by others in 24-h cross-over studies (18, 19). These two investigations reported time in the target range and time >98% during A-FiO_2_, which were comparable to our findings. However, they reported that the exposure to SpO_2 _< 80% was less than 2%, similar to what we found in our stable cohort but markedly less than our less-stable cohort. Although small, a difference of 1% time with SpO_2 _< 80% is definitely of potential clinical relevance and thus important (2). The difference in target range between studies is certainly a factor in the increased exposure to hypoxemia in our study. We also suggest that the timing of the selection of subjects for short-term cross-over studies is also highly relevant. The infants in these reports were studied on A-FiO_2_ for only one day and generally later in life. They were also selected considering their stability for a 48 h cross-over period. We do not discount the importance of structured manual vs. automated short cross-over studies in evaluating the potential of A-FiO_2_. However, we believe that assessing the treatment across the continuum of care is not only more reflective of what can be expected with routine use but also essential to considering the potential impact on neonatal outcomes.

As with all medical devices, it is also important that they are used optimally. The performance of A-FiO_2_ systems has been consistent in numerous studies across a range of different devices (7). New systems, or system enhancements, must be thoroughly evaluated before being routinely used. Determining optimal practices is particularly relevant when introducing new technologies such as A-FiO_2_. Such studies have thoroughly evaluated different target ranges (20–22). Explorations of other subtleties are limited. One study evaluated different alarm strategies (13) and another evaluated different FiO_2_ adjustment rates (14). Further variation in clinical approaches to use, often seen in pragmatic studies, may mitigate potential benefits or risks.

This study has some limitations. While it reflects routine use in our unit, there is insufficient experience with intubated infants (conventional and HFOV) and infants with BPD. Thus, these findings should be applied with caution to those modes of support that are more common in the first 2 weeks of life. There are seven averaging time settings available with the Masimo oximeter, and we only tested three. However, these cover the range of the seven settings, and our results showed that longer is better. Consistent with our subjective impression, the system works well with these three set SpO_2_ averaging time settings, and the differences we found were small and perhaps not clinically relevant. Further, while the consensus is that the short averaging periods are most reflective of clinical exposure, this may not be the case (14). They typically reflect only three peripheral pulses and may be less clinically relevant than a more prolonged average when considering measurement noise and duration. Finally, our findings apply to only one A-FiO_2_ device.

## Conclusion

This A-FiO_2_ algorithm is effective regardless of the SpO_2_ averaging time setting. There appears to be an advantage of a small decrease in exposure to SpO_2_ extremes associated with longer settings. This suggests there is an interaction between the averaging setting and the controller.

Our unit changed the default setting to 10 s, with an option for the attending physician to adjust if necessary. More research is needed to explore the optimal use of A-FiO_2_ systems, whether in the context of CQI or controlled studies.

## Data Availability

The raw data supporting the conclusions of this article will be made available by the authors without undue reservation.
